# Reconstruction of scalp defects with the radial forearm free flap

**DOI:** 10.1186/1758-3284-4-21

**Published:** 2012-05-14

**Authors:** Larissa Sweeny, Brendan Eby, J Scott Magnuson, William R Carroll, Eben L Rosenthal

**Affiliations:** 1 Department of Surgery, Division of Otolaryngology – Head and Neck Surgery, University of Alabama at Birmingham, Alabama, USA; 2 Division of Otolaryngology, BDB Suite 563, 1808 7th Avenue South, AL, 35294-0012, Alabama, USA

**Keywords:** Scalp defect, Free flap, Calvarium, Reconstruction, Cancer

## Abstract

**Background:**

Advanced and recurrent cutaneous squamous cell carcinoma of the scalp and forehead require aggressive surgical excision often resulting in complex defects requiring reconstruction. This study evaluates various microvascular free flap reconstructions in this patient population, including the rarely utilized radial forearm free flap.

**Patients and methods:**

A retrospective review of patients undergoing free flap surgeries (n = 47) of the scalp between 1997 and 2011 were included. Patients were divided primarily into two cohorts: a new primary lesion (n = 21) or recurrence (n = 26). Factors examined include patient demographics, indication for surgery, defect, type of flap used, complications (major and minor), and outcomes.

**Results:**

The patients were primarily male (n = 34), with a mean age of 67 years (25–91). A total of 58 microvascular free flap reconstructions were performed (radial forearm free flap: n = 28, latissimus dorsi: n = 20, rectus abdominis: n = 9, scapula: n = 1). Following reconstruction with a radial forearm free flap, duration of hospitalization was shorter (*P* = 0.04) and complications rates were similar (*P* = 0.46). Donor site selection correlated with defect area (*P* < 0.001), but not with the extent of skull defect (*P* = 0.70). Larger defect areas correlated with higher complications rates (*P* = 0.03) and longer hospitalization (*P* = 0.003). Patients were more likely to require multiple reconstructions if referred for a recurrent lesions (*P* = 0.01) or received prior radiation therapy (*P* = 0.02).

**Conclusion:**

Advanced and recurrent malignancies of the scalp are aggressive and challenging to treat. The radial forearm free flap is an underutilized free flap in the reconstruction of complex scalp defects.

## Introduction

The advent of free tissue transfer to the field of head and neck reconstructive surgery has greatly expanded the repertoire of techniques available for treating defects of the scalp and forehead [1,2]. Free flap reconstruction has rapidly established itself as a desirable and versatile therapy for defects of the scalp, especially in larger and more complex cases [3,4]. Scalp defects secondary to surgical resection of a cutaneous malignancy may necessitate reconstruction with a microvascular free flap. Indications for microvascular free flap reconstruction include but are not limited to: defect size preventing primary closure, failed primary or local flap closure due to inelastic or poor skin quality, multiple resections for recurrences, or neo- or adjuvant radiation therapy.

**Figure 1:**
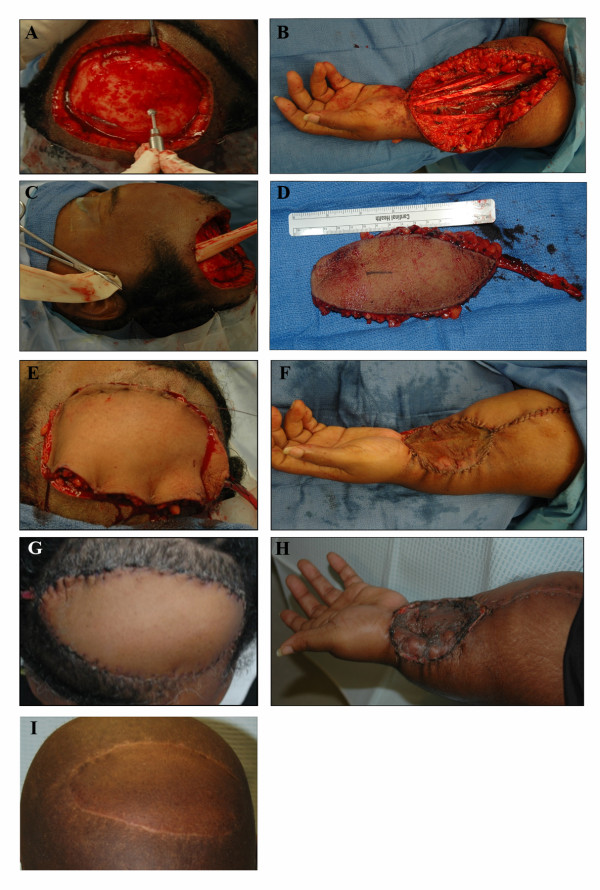
**A 50 year old female presented with recurrent, poorly differentiated squamous cell carcinoma**. A radical excision of the lesion, including removal of the outer table, was performed (**A**). A subfascial dissection of the radial forearm flap was performed with preservation of the radial nerve (**B**). A subcutaneous tunnel was created to access the temporal vessels (**C**). The harvested radial forearm free flap measured 6 x 12 cm (**D**). The radial forearm free flap was inset in the scalp over a drain using interrupted sutures (**E**). A split thickness skin graft harvested from the thigh was used to cover the donor site (**F**). Two weeks post-reconstruction the recipient and donor sites were healthy without evidence of flap or graft loss (G, H). Following radiation therapy the radial forearm free flap was well healed and maintained good contour (**I**)

The advantage and effectiveness of microvascular free flap reconstructive techniques depend on various factors including etiology and size of the defect, donor site morbidity, and involvement of surrounding structures. A number of articles have discussed microvascular free flap reconstruction of complex scalp defects following calvarial and dural resections [5-8]. However, previous publications report limited utilization of the radial forearm free flap (< 10%), if any, for reconstructions of the scalp. Advantages of the radial forearm free flap include the ability to harvest without having to reposition the patient and shorter operative times (Figure 1). The purpose of this article is to present our experiences with microvascular free flap reconstruction of scalp defects and compare various microvascular free flap selections.

## Patients and methods

### Patient selection

Following Institutional Review Board approval, a retrospective chart review was performed. Patients who had a cutaneous scalp malignancy requiring resection and a microvascular free flap reconstruction consecutively managed at the University of Alabama at Birmingham between January 2001 and December 2011 were included (n = 47). Indications for surgical intervention included advanced stage primary (n = 21) or recurrent (n = 26) cutaneous malignancy of the scalp. Lesions restricted to the auricular-temporal region and requiring a mastoidectomy were excluded. Tumor histology was confirmed by the pathology department.

### Statistical analyses

Descriptive variables were summarized by mean (± SD) for continuous variables and %, n for categorical variables. A student’s *t*-test was used to compare differences in means between groups. A one-way ANOVA was used to analyze relationships between categorical factors and continuous responses. A contingency analysis was used to analyze relationships between categorical factors and responses. Univariate analyses were performed using the Pearson bivariate correlation coefficient. Multivariate analyses were performed to analyze the combined effects of multiple variables on various outcomes. For analyses comparing type of free flap reconstruction, the patient who received a scapular free flap reconstruction (n = 1) was excluded due to low sample size. A *P*-value of < 0.05 was considered statistically significant. Statistical analysis was performed using Jmp 9.0.2 software (SAS, Cary, NC).

## Results

### Patient and scalp defect characteristics

The mean age of the patients was 66.7 (25–91) with the majority of the patients being male (72%, n = 34). The vast majority of patients were Caucasian (91%, n = 43), with the remaining being Black (9%, n = 4). The most common pathology was squamous cell carcinoma (57%, n = 27), followed by sarcoma (19%, n = 9) and basal cell carcinoma (17%, n = 8). While only 9% had a history of prior radiation therapy, 40% received post-reconstruction adjuvant radiation therapy (n = 19). Thirty-eight percent had a history of pre-operative tobacco usage (n = 18) and tobacco usage was not associated with the surgical indication (primary or recurrent lesion) (*P* = 0.93, R^2^ = 0.0001). At the time of initial microvascular free flap operation, 19% were immunocompromised (n = 9), 52% had cardiac disease, 19% had diabetes mellitus and 19% had a history of an additional non-cutaneous malignancy (Table 1). Cardiac disease included patients with hypertension (n = 22), coronary artery disease (n = 5), prior heart transplant (n = 1) and cardiomyopathy (n = 1). Immunocompromised included prior organ transplant patients on immunosuppressive therapy (n = 5) and those patients on corticosteroids (n = 4).

**Table 1:** Patient characteristics

**Characteristic**	**% (n = 47)**
**Age** (range)	66.7 (25–91)
**Gender**	
Male	72 (34)
Female	28 (13)
**Race**	
Caucasian	91 (43)
Black	9 (4)
**Surgical Indication**	
Primary Lesion	45 (21)
Recurrent Lesion	53 (26)
**Prior Radiation Therapy**	
No	91 (43)
Yes	9 (4)
**Adjuvant Radiation Therapy**	
No	60 (28)
Yes	40 (19)
**Tobacco History**	
No	62 (29)
Yes	38 (18)
**Medical Comorbidities**	
Immunocompromised	19 (9)
Cardiac Disease	52 (24)
Diabetes Mellitus	19 (9)
History of an additional non-cutaneous malignancy	19 (9)

^*^ Other tumor histologies include: eccrine carcinoma, melenoma, and merkel cell carcinoma.

Every scalp defect was the result of an oncologic resection and each patient underwent at least one microvascular free flap reconstruction for the defect. The frontal region of the scalp was the most common defect location (31%, n = 18), followed by the occipital (24%, n = 14), parietal (19%, n =11), temporal (14%, n = 8) and temporal-parietal (12%, n = 7) regions (Table 2). The mean area of the defect was 135 cm^2^ (±125). When broken down by region, the defects with the largest areas were located in the occipital region with the only statistical difference being between occipital and frontal regions (*P* = 0.028) (Table 3). Over half of the reconstructions were for defects involving calvarium resection [23% outer table (n = 11) or 30% craniectomy (n = 14)] and 20% had a durotomy (n = 12) (Table 2). Only one calvarial defect was reconstructed and titanium mesh was used, while all other durotomies were repaired with a duraplasty. Those patients requiring a craniectomy and durotomy had significantly larger defect surface areas compared to craniectomy alone (*P* = 0.001), outer table only (*P* = 0.0005) or no cranial excision (*P* = 0.0003) (Table 3). All patients with a history of prior radiation therapy required a calvarium resection, and as a result, there was a significant relationship between a history of prior radiation therapy and calvarium resection (*P* = 0.017, R^2^ = 0.11). The mean duration of hospitalization was 6.1 days (±3.6). The location of the defect (*P* = 0.41) or a calvarium defect (*P* = 0.27) did not significantly affect the duration of hospitalization (Table 4).

**Table 2:** Free flap reconstruction of scalp defects

	**Total**	**Latissimus dorsi**	**Rectus abdominis**	**Radial forearm**	**Scapula**
	**% (n = 58)**	34 (n = 20)	15 (n = 9)	47 (n = 28)	2 (n = 1)
**Defect Location**					
Frontal	31 (18)	15 (3)	33 (3)	43 (12)	-
Parietal	19 (11)	35 (7)	22 (2)	7 (2)	-
Temporal	14 (8)	5 (1)	11 (1)	21 (6)	-
Temporal-Parietal	12 (7)	5 (1)	22 (2)	14 (4)	-
Occipital	24 (14)	40 (8)	11 (1)	14 (4)	100 (1)
**Cranial Defect**					
None	38 (22)	40 (8)	33 (3)	36 (10)	100 (1)
Outer Table	28 (16)	20 (4)	22 (2)	36 (10)	-
Craniectomy	34 (20)	40 (8)	44 (4)	29 (8)	-
+ Dural Excision	20 (12)	25 (5)	44 (4)	11 (3)	-
**Surgical Complications**					
None	60 (35)	50 (10)	44 (4)	72 (20)	-
Intervention Required	40 (23)	45 (9)	56 (5)	28 (8)	100 (1)
Multiple	19 (11)	20 (4)	44 (4)	7 (2)	100 (1)
**Mean age (range)**	58 (25–91)	72 (56–91)	67 (53–88)	64 (25–87)	45 (−)

**Table 3:** **Mean surface area (cm**^**2**^**) of the scalp defect**

	**Total**	**Latissimus dorsi**	**Rectus abdominis**	**Radial forearm**	**Scapula**
	**mean (± SD)**	211 (163)	203 (133)	72 (35)	24 (−)
**Defect Location**					
Frontal	101 (68)	180 (0)	195 (13)	57 (22)	-
Parietal	113 (63)	122 (77)	138 (59)	93 (4)	-
Temporal	184 (169)	330 (−)	540 (−)	101 (43)	-
Temporal-Parietal	98 (47)	150 (−)	123 (38)	73 (42)	-
Occipital	203 (198)	300 (213)	180 (−)	61 (33)	24 (−)
**Cranial Defect**					
None	111 (74)	146 (98)	152 (49)	79 (35)	24 (−)
Outer Table	108 (78)	194 (84)	175 (35)	60 (27)	-
Craniectomy	195 (186)	284 (220)	255 (196)	77 (42)	-
+ Dural Excision	268 (209)	392 (210)	255 (196)	79 (38)	-
**Surgical Complications**					
None	114 (79)	158 (113)	164 (46)	80 (34)	-
Intervention Required	192 (178)	275 (197)	234 (176)	51 (28)	24 (−)
Multiple	172 (139)	178 (21)	268 (183)	46 (5.7)	24 (−)

**Table 4:** Mean length of hospital admission (days)

	**Total**	**Latissimus dorsi**	**Rectus abdominis**	**Radial forearm**	**Scapula**
	**mean (± SD)**	7.6 (3.3)	9 (4.6)	5.8 (3.2)	5 (−)
**Defect Location**					
Frontal	8.2 (5.1)	7.5 (0.7)	13.7 (4.9)	6.3 (4.3)	-
Parietal	7.1 (3.0)	9.4 (3.7)	5.5 (2.1)	4 (0)	-
Temporal	5.7 (1.9)	- (−)	8 (−)	5.3 (1.8)	-
Temporal-Parietal	6.4 (3.0)	5 (−)	6.5 (3.5)	6.8 (3.6)	-
Occipital	6 (2.1)	6.3 (2.6)	8 (−)	5.3 (1.0)	5 (−)
**Cranial Defect**					
None	5.7 (1.9)	5.9 (2.0)	7 (2.6)	5.3 (1.7)	5 (−)
Outer Table	7.3 (5.1)	8.8 (5.0)	10 (8.5)	6.1 (4.7)	-
Craniectomy	7.9 (3.4)	8.6 (2.9)	10 (4.7)	6.1 (2.5)	-
+ Dural Excision	7.9 (3.4)	7.8 (2.4)	10 (4.7)	5.3 (0.6)	-
**Surgical Complications**					
None	5.8 (2.4)	6.5 (1.4)	8.8 (5.1)	4.9 (1.3)	-
Intervention Required	8.6 (4.4)	8.9 (4.3)	9.2 (4.8)	8.3 (5.0)	5 (−)
Multiple	8.5 (4.6)	9.8 (5.9)	9.0 (5.0)	6.5 (2.1)	5 (−)

### Comparison of microvascular free flap reconstructive approaches

The most common free flap used for reconstruction of the scalp was a radial forearm fasciocutaneous free flap (48%, n = 28; Figure 1), followed by a latissimus dorsi muscle free flap and split thickness skin graft (34%, n = 20) and rectus abdominis myocutaneous free flap (16%, n = 9). Only one patient was reconstructed with a scapular free flap (2%). The mean age of patients who were reconstructed with radial forearm free flap (64.4) was significantly younger than those reconstructed with a latissimus dorsi free flap (71.8) (*P* = 0.046). Nine patients (19%) underwent more than one free flap reconstruction for either multiple primaries (n = 2), re-excision of a recurrence (n = 5), venous congestion resulting in flap failure (n = 1), chronic wound infection resulting in complete-flap failure (n = 1), or chronic wound infection resulting in partial-flap failure (n = 2). Patients requiring multiple microvascular free flap reconstructions for indications other than a second primary (n = 2) were compared to those requiring a single reconstruction only. Only patients whom presented to our institution for management of a recurrent lesion required multiple reconstructions (n = 7, *P* = 0.012, R^2^ = 0.24). These patients had all undergone a previous surgical excision at an outside hospital. In addition, patients who received radiation therapy were more likely to require multiple microvascular free flap reconstructions (*P* = 0.023, R^2^ = 0.16).

When comparing latissiumus dorsi (n = 20), rectus abdominis (n = 9) and radial forearm free flaps (n = 28), location of defect significantly affected type of microvascular free flap used for the reconstruction (*P* = 0.048, R^2^ = 0.09). The majority of the latissiumus dorsi free flaps were used to reconstruct defects of the occipital (40%, n = 8) or parietal region (35%, n = 7), while the majority of radial forearm free flaps were used to reconstruct defects of the frontal region (43%, n = 12). The type of cranial defect, however, did not affect the microvascular free flap selected for reconstruction (*P* = 0.35, R^2^ = 0.07) (Table 2). The surface area of the defect did affect which microvascular free flap was selected for the reconstruction (*P* < 0.0001, R^2^ = 0.28). The mean area (cm^2^) of the defect was significantly smaller for the radial forearm (72 cm^2^) than latissimus dorsi (211 cm^2^; *P* < 0.0001) or rectus abdominis (203 cm^2^; *P* = 0.0034) (Table 2). Furthermore, the microvascular free flap used for the reconstruction was found on multivariate analyses to be significantly affected by the region and surface area of the defect (*P* < 0.0001, R^2^ = 0.60), and by the defect surface area and extent of cranial excision (*P* < 0.0001, R^2^ = 0.36) (Table 3).

The mean duration of hospitalization (days) was shortest for those patients reconstructed with a radial forearm free flap (5.8 days), followed by latissimus dorsi free flap (7.6 days) and rectus abdominis free flap (9.0 days) (*P* = 0.04, R^2^ = 0.11). When analyzed by donor site, the only statistical differences in duration of hospitalization were between radial forearm and rectus free flaps (*P* = 0.02), although the difference between radial forearm and latissimus dorsi free flaps trended toward significance (*P* = 0.085) (Table 4). Additional multivariate analyses found the type of microvascular free flap used for the reconstruction and either the location of the defect (*P* = 0.091, R^2^ = 0.35) or cranial defect (*P* = 0.32, R^2^ = 0.21) did not affect the length of hospitalization.

### Postoperative complications

Eighteen patients underwent operative intervention for management of a complication or for aesthetic improvement (Table 5). Indications for reoperation included: donor site hematoma requiring evacuation (n = 5), recipient site hematoma requiring evacuation (n = 3), wound debridement (n = 5), fistula excision (n = 1), abdominal repair (n = 1), scar revision (n = 2), flap debulking (n = 5), and brow lift (n = 1). In addition, one patient required leech therapy for venous insufficiency of a radial forearm free flap. There was a higher incidence of donor site hematomas following reconstruction with a latissimus dorsi (20%, n = 5) than a radial forearm (4%, n = 1) free flap. Similarly, there was a higher incidence of recipient site hematomas following reconstruction with a rectus abdominis (22%, n = 2) compared to the radial forearm (7%, n = 2) free flap. The n for each group was not significant enough to draw any statistical conclusions. There was one perioperative death in a patient whom underwent a craniectomy and durotomy and subsequently suffered a subdural intraventricular hemorrhage and myocardial infarction on post-operative day 8.

**Table 5:** Microvascular free flap reconstruction complications requiring operative intervention

	**Total**	**Latissimus dorsi**	**Rectus abdominis**	**Radial forearm**	**Scapula**
	**40% (n = 23/58)**	45 (9/20)	56 (5/9)	29 (8/28)	100 (1/1)
**Donor Site**					
Hematoma	9 (5)	20 (4)	-	4 (1)	-
Debridement	2 (1)	5 (1)	-	-	-
Dehiscence	2 (1)	-	11 (1)	-	-
**Recipient Site**					
Hematoma	10 (6)	-	22 (2)	7 (2)	100 (1)
Debridement	5 (3)	5 (1)	11 (1)	4 (1)	-
Dehiscence	5 (3)	-	11 (1)	7 (2)	-
Venous Insufficiency	2 (1)	-	-	4 (1)	-
Necrosis	2 (1)	5 (1)	-	-	-
Fistula	2 (1)	-	-	4 (1)	-
Failure	3 (2)	10 (2)	-	-	-

The occurrence of a surgical complication was not affected by the regional location of the scalp defect (*P* = 0.21, R^2^ = 0.08), extent of cranial excision (*P* = 0.47, R^2^ = 0.02), prior radiation therapy (*P* = 0.52, R^2^ = 0.005), adjuvant radiation therapy (*P* = 0.97, R^2^ = 0.002), multiple microvascular free flap reconstructions (*P* = 0.11, R^2^ = 0.03), gender (*P* = 0.17, R^2^ = 0.03), race (*P* = 0.54, R^2^ = 0.005), being immunocompromised (*P* = 0.66, R^2^ = 0.002), having cardiac disease (*P* = 0.07, R^2^ = 0.04), having diabetes mellitus (*P* = 0.66, R^2^ = 0.002) or having a history of another non-cutaneous malignancy (*P* = 0.66, R^2^ = 0.03). However, the occurrence of a surgical complication was affected by age (*P* = 0.017, R^2^ = 0.10). The mean age was higher for those patients who had a complication (73, n = 23) compared to none (63, n = 35) (*P* = 0.006, R^2^ = 0.13). A trend toward greater complications rates was found in more males (44%, n = 20) than females (23%, n = 3) (*P* = 0.17, R^2^ = 0.03) and in patients presenting with a recurrent lesion (47%, n = 15) compared to a primary lesion (22%, n = 5) (*P* = 0.18, R^2^ = 0.03).

The mean defect area was significantly larger in those patients who had a complication (192 cm^2^) compared to those that had no complications (114 cm^2^) (*P* = 0.033). Furthermore, the area of the defect had a significant effect on the occurrence of a surgical complication (*P* = 0.033, R^2^ = 0.06) (Table 3). The mean duration of hospitalization was significantly longer for patients when a complication occurred (8.6 days) compared no complication (5.8 days, *P* = 0.003) (Table 4).

When comparing surgical complications, there was a higher incidence of a complication following reconstruction with a latissiumus dorsi (45%, *P* = 0.27) or rectus abdominis (56%, *P* = 0.14) free flap compared to a radial forearm (29%) free flap. The incidence of surgical complications was found on multivariate analysis to be significantly affected by the type of microvascular free flap (latissiumus dorsi, rectus abdominis, radial forearm) used for the reconstruction, region of the scalp being reconstructed, type of cranial defect, pre-operative tobacco usage, prior radiation therapy and a history of cardiac disease (*P* = 0.015, R^2^ = 0.93). Additional multivariate analysis found the type of microvascular free flap (latissiumus dorsi, rectus abdominis, radial forearm) used for the reconstruction, type of cranial defect, preoperative tobacco usage, history of cardiac disease and the surgical complication affected the length of hospitalization (*P* = 0.0016, R^2^ = 0.89).

## Discussion

Advanced and recurrent cutaneous malignancies of the scalp often require extensive resection to achieve adequate margins, resulting in a complex scalp defect. Several characteristics of the scalp contribute to the reconstructive challenges of these defects. These include tissue inelasticity, the convex shape of the cranium, involvement of the cranium requiring composite resection, and the remoteness from proximal flaps. In addition, these patients often require adjuvant radiation therapy, necessitating durable coverage and swift healing to accommodate therapy start times [3]. Several factors affect the selection of a microvascular free flap including location of the defect, size of the defect, prior reconstructions, patient age and body habitus, donor-site morbidity, depth of the defect and surgeon preference [9]. In our review of the literature, the vast majority of large scalp defects have been reconstructed with a latissimus dorsi free flap (49%, n = 280/567), followed by rectus abdominis (17%, n = 96/567) and anterior lateral thigh (14%, n = 77/567). Surprisingly, the radial forearm free flap was rarely used (8%, 44/567) [1-24].

Even in publications from the past 15 months, the radial forearm free flap remains an uncommon choice for microvascular reconstruction of major scalp defects (4%, n = 9/204) [6-8,23,24]. In contrast, at our institution we have found an increasing niche for the radial forearm free flap. Previous investigators have cited reasons for choosing latissimus dorsi or anterior lateral thigh over other free tissue transfers to be their long vascular pedicle, large surface area, high vascularization, ease of harvest and low donor site morbidity [12,15,20,24-26]. We feel the radial forearm also has several advantages including decreased positioning needs, greater ease of harvest, low donor site morbidities and a reliable vascular supply as well. In the current study we found no statistical advantage of any one microvascular free flap over the others and our microvascular free flap failure rate (3.4%, n = 2) is comparable to other studies [3,7].

The average size of the defect (135 cm^2^) was also similar to previous publications [2,5,12]. The defects with the largest surface areas were located in the occipital region with the only statistical difference being between occipital and frontal regions (*P* = 0.028). Those patients requiring a craniectomy and durotomy had significantly larger defect surface areas compared to craniectomy alone (*P* = 0.001), outer table only (*P* = 0.0005) or no cranial excision (*P* = 0.0003). The mean duration of hospitalization (6.1 days) was not affected by the size of defect (*P* = 0.17), location of the defect (*P* = 0.41) or extent calvarium resection (*P* = 0.27). However, the mean duration of hospitalization did vary by free flap reconstruction: radial forearm free flap (5.8 days), followed by latissimus dorsi free flap (7.6 days) and rectus abdominis free flap (9.0 days) (*P* = 0.04).

There are institutional variations in regards to whether the calvarium is reconstructed following a composite resection [5,7,8]. At our institution, it is not common for the calvarium to be reconstructed following composite resection. It has been our experience that cranioplasty materials are at a high risk for exposure, especially following post-operative radiation therapy. Other authors have cited a potential increased risk of morbidity and mortality without the protection normally provided by the cranium or cranioplasty [7]; however this has not been found at our institution to be the case. Similar to previous publications we found no difference in complication rates for patients who underwent calvarium resection in addition to their scalp resection (*P* = 0.81) [7].

The mean age of patients who were reconstructed with radial forearm free flap (64.4) was significantly younger than those reconstructed with a latissimus dorsi free flap (71.8) (*P* = 0.046). In addition, the mean area of the defect was significantly smaller for the radial forearm (72 cm^2^) than latissimus dorsi (211 cm^2^; *P* < 0.0001) or rectus abdominis (203 cm^2^; *P* = 0.0034). The location of the scalp defect also significantly affected which microvascular free flap was selected for the reconstruction (*P* = 0.048). The majority of latissiumus dorsi free flaps were used to reconstruct defects of the occipital (40%) or parietal region (35%), while the majority of radial forearm free flaps were used to reconstruct defects of the frontal (43%) or temporal region (21%). The extent of the cranial defect, however, did not affect the microvascular free flap used for reconstruction (*P* = 0.35). Therefore, in patients with a scalp defect larger than 7 cm in width or older patients whose forearm skin has thinned and lacks adequate elasticity, a latissimus dorsi free flap is recommended. However, in cases of smaller defects or defects located in the frontal or temporal region of the scalp, the radial forearm free flap is preferable given its shorter operative times and duration of hospitalization.

When comparing complications requiring surgical intervention, there was a higher incidence of a complication following reconstruction with a latissiumus dorsi (45%, *P* = 0.27) or rectus abdominis (56%, *P* = 0.14) free flap compared to a radial forearm (29%) free flap. Furthermore, the temporal-parietal (57%) and occipital (54%) regions were associated with higher incidences of complications, followed by the frontal (39%), parietal (27%) and temporal (12.5%) regions. Confounding risk factors for a complication included the type of microvascular free flap used for the reconstruction, region of the scalp being reconstructed, type of cranial defect, pre-operative tobacco usage, prior radiation therapy and a history of cardiac disease (*P* = 0.015). Additionally, the duration of hospitalization was affected by the type of microvascular free flap used for the reconstruction, type of cranial defect, preoperative tobacco usage, history of cardiac disease and the surgical complication (*P* = 0.0016, R^2^ = 0.89).

Only those patients whom presented to our institution for management of a recurrent lesion required multiple reconstructions (*P* = 0.012). These patients had all undergone a previous surgical excision at an outside hospital. Further analysis found previous radiation therapy increased the likelihood of requiring multiple reconstructions (*P* = 0.023). Of the 9 patients who underwent multiple reconstructions, 5 were for excision of a recurrence. Consequently, these patients likely have a more aggressive disease pathogenesis which may be contributing to an increased rate of reconstruction.

## Conclusions

The radial forearm free flap is an underutilized option for reconstruction of complex scalp defects. The radial forearm free flap was associated with few complications requiring surgical intervention and shorter duration of hospitalization. Younger patients and patients with smaller defects (≤ 7 cm in width) were more likely to be reconstructed with a radial forearm free flap. In addition, the radial forearm free flap was used more commonly for reconstructions of defects in the frontal region. The selection of microvascular free flap used in the reconstruction was not affected by the extent of the cranial defect. Surgical intervention for a recurrent lesion and history of prior radiation therapy correlated with patients requiring multiple microvascular free flap reconstructions.

## This work was presented at the annual Combined Otolaryngology Spring Meeting to be held in San Diego, April 20, 2012

## Competing interests

The authors have no competing of interest to disclose.

## Authors’ contributions

LS carried out data collection, data analysis, statistical analysis, and composition of the manuscript. BE carried out project inception and design as well as data collection. JSM provided data for the analysis. WRC provided data for the analysis and edited the final manuscript. ELR carried out project inception and design, data analysis, manuscript composition, and manuscript editing. All authors read and approved the final manuscript.

## Financial disclosures

This work was supported by the National Institutes of Health/National Cancer Institute grants 2 T32 CA091078-09.
